# Hypersensitivity Pneumonitis Associated with Environmental Mycobacteria

**DOI:** 10.1289/ehp.7727

**Published:** 2005-02-10

**Authors:** William Beckett, Michael Kallay, Akshay Sood, Zhengfa Zuo, Donald Milton

**Affiliations:** ^1^Pulmonary and Critical Care Division, Occupational Medicine Program and Finger Lakes Occupational Health Services, University of Rochester School of Medicine and Dentistry, Rochester, New York, USA;; ^2^Division of Pulmonary and Critical Care Medicine, Southern Illinois University School of Medicine, Springfield, Illinois, USA;; ^3^Harvard School of Public Health, Brookline, Massachusetts, USA

**Keywords:** atypical mycobacteria, extrinsic allergic alveolitis, hot tub, hypersensitivity pneumonitis, metalworking fluids, *Mycobacterium chelonae*, *Mycobacterium immunogenum*, nontuberculous mycobacteria, occupational lung disease

## Abstract

A previously healthy man working as a machine operator in an automotive factory developed respiratory symptoms. Medical evaluation showed abnormal pulmonary function tests, a lung biopsy showed hypersensitivity pneumonitis, and his illness was traced to his work environment. His physician asked the employer to remove him from exposure to metalworking fluids. Symptoms reoccurred when he was later reexposed to metalworking fluids, and further permanent decrement in his lung function occurred. Investigation of his workplace showed that five of six large reservoirs of metalworking fluids (cutting oils) grew *Mycobacterium chelonae* (or *Mycobacterium immunogenum*), an organism previously associated with outbreaks of hypersensitivity pneumonitis in automaking factories. His lung function remained stable after complete removal from exposure. The employer, metalworking fluid supplier, union, and the National Institute for Occupational Safety and Health were notified of this sentinel health event. No further cases have been documented in this workplace.

## Case Presentation

**Figure 1:**
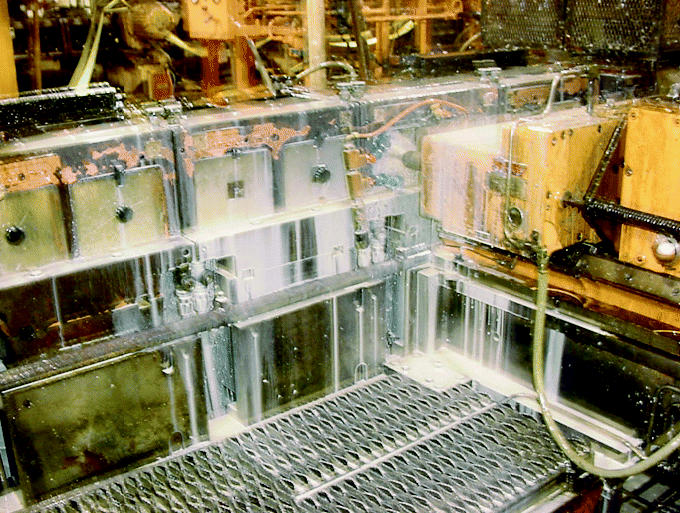
In a process similar to that used by the patient, metalworking fluid (milky appearance) is flowed over auto parts to reduce friction and cool metal tools. As fluids are sprayed over metal parts, a visible aerosol is formed that can be breathed by operators of the machinery unless specific control measures are instituted. Fluids are recycled from large holding tanks. The presence of carbon and water in fluids permits growth of microorganisms, including mycobacteria.

A 57-year-old nonsmoking auto-parts machine operator presented in 1995 because of shortness of breath on exertion, cough, fatigue, and chest congestion. In his job he operated a machine that cut metal parts using a semi-synthetic metalworking fluid (Figure 1) that was collected and recycled through large tanks holding > 1,000 gal fluid. A chest radiograph showed a generalized increase in interstitial markings. He was treated with empiric antibiotics on two occasions. Later, his treating physician suspected occupational asthma due to exposure to oil mist, and asked the employer to remove him from exposure to metalworking fluids. A trial of bronchodilator medications was not effective in improving his symptoms, which were worse after work. Spirometry was performed by the factory’s medical department just before and after a 5-day work week; no change in spirometry was noted. A measurement of total metalworking fluid aerosol done in the patient’s work area showed that the mass of aerosol was 0.42 mg/m^3^ of air sampled, which was below the recommended limit of a recent advisory committee.

**Figure 2:**
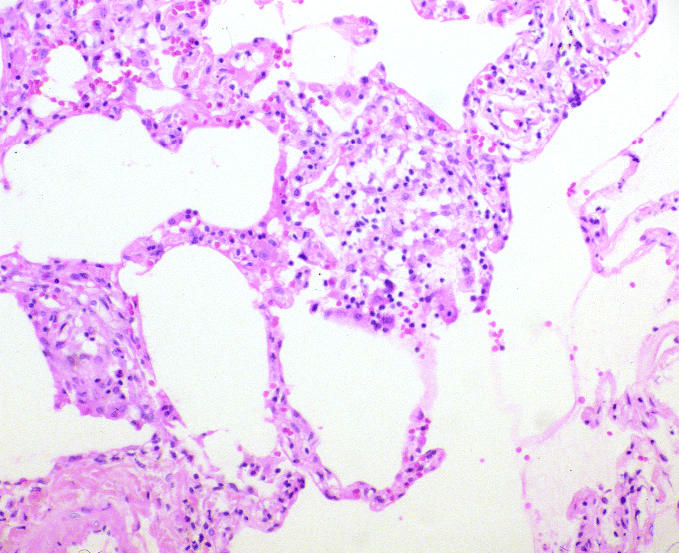
Transbronchial biopsy specimen of the patient’s lung showing marked alveolar inflammation and cell proliferation with the presence of inflammatory and epithelioid cells.

When the physician’s recommendations to remove the patient from all metalworking fluids was not followed and symptoms persisted, the patient was referred to a pulmonary specialist for further testing. Pulmonary function tests showed a reduced diffusing capacity of 67% predicted with oxygen desaturation on ambulation (Table 1), and a carbachol challenge (a test for airway hyperreactivity in asthma) was negative. Bronchial alveolar lavage showed 90% lymphocytes and 10% macrophages in alveolar lining fluid, with negative smear and culture for acid fast bacilli (mycobacteria) and fungi. A transbronchial lung biopsy (Figure 2) showed interstitial chronic inflammation and collections of epithelioid cells suggestive of granulomas with negative stains for acid-fast bacilli and fungus and, on review, diagnostic of hypersensitivity pneumonitis. Several years later, testing of the preserved tissue block by polymerase chain reaction was negative for sequences found in *Mycobacterium chelonae*. The patient’s treating pulmonologist suspected that the hypersenstivitiy pneumonitis was due to bacteria growing in the metalworking fluid. Serum-precipitating antibodies to a standard panel of nine substances, including bacteria, several fungi, and pigeon serum, were all negative. The pulmonologist gave the patient a brief note for his employer restricting exposure to metalworking fluids; the company physician misinterpreted the message as indicating that the patient had chronic obstructive pulmonary disease made worse by metalworking fluid exposures, and changed his work location but did not fully restrict him from exposure to metalworking fluids.

**Figure 3:**
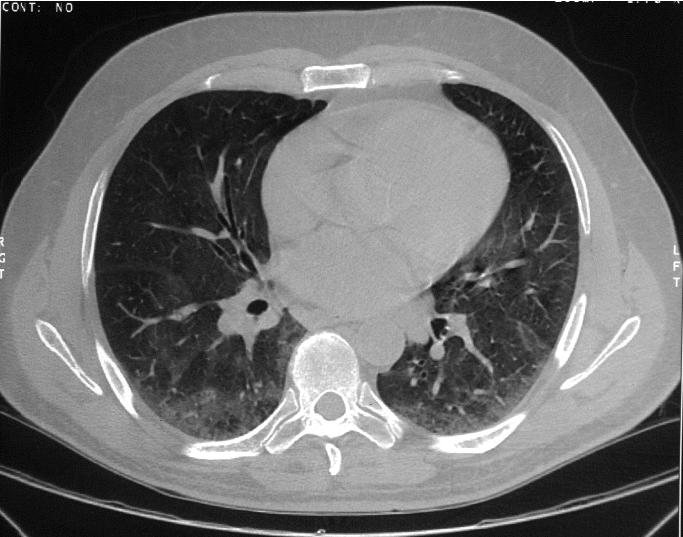
Thin-section CT scan of the chest showing ground glass opacities in the lung parenchyma, indicating interstitial inflammation and/or fibrosis.

No specific interventions were made in the workplace with regard to the metalworking fluids, although a plantwide program of reduction of fluid aerosol exposures for all workers was already in progress. Several months later the patient had an uncomplicated myocardial infarction, and after 3 months returned to work with continued exposure to metalworking fluids. Three years later, in 2000, he noted daily nasal congestion associated with work, and worsening dyspnea on exertion. His pulmonologist repeated lung function tests, which showed a further decline in diffusing capacity to 44% predicted (Table 1), and a thin-section computed tomography (CT) scan of the chest (Figure 3) showed “ground glass” opacities indicating interstitial lung disease and mild bronchiectasis. A visit to the patient’s residence by a treating physician trained in occupational and environmental medicine did not reveal any exposures suggestive of contributing to his hypersensitivity pneumonitis.

With the assistance of the county health department, samples of metalworking fluid were obtained for culture from the large reservoir supplying metalworking fluids to the patient’s work area. Standard bacterial and fungal counts were below the level of detection of 10 organisms/mL, unusually low for industrial metalworking fluids, which are usually contaminated by microorganisms. Stain of the centrifuged fluid pellet for acid-fast bacilli was qualitatively “very high,” and culture grew 1.6 × 10^5^ mycobacteria/mL, which were identified as *M. chelonae*. This mycobacterium, although similar to the *M. chelonaeabscessus* group, has been proposed as a new species, *Mycobacterium immunogenum* (Brown-Elliott and Wallace 2002). Additional, separate fluid specimens were sent to another laboratory, which cultured and identified the same organism. Samples of fluid from five reservoirs, a blank of “virgin” metalworking fluid, and a tap water control were for a third time tested and showed > 2,500 mycobacteria/mL, with single-stranded conformational polymorphism analysis showing *M. chelonae* subtype *M. immunogenum* in the used fluid samples, and none in the virgin fluid or tap water. Endotoxin, the active agent in the walls of gram-negative bacteria, was measured in the five samples from the five reservoirs at from 2.4 × 10^2^ to 2.5 × 10^4^ endotoxin units per milliliter of fluid by the *Limulus* assay. Based on these findings, the patient was removed completely from exposure to metalworking fluids.

The treating occupational physician scheduled a meeting with the plant occupational physician, industrial hygienists, and the contracting supplier of the metalworking fluids to recommend *a*) a survey of symptoms and chest X rays of workers exposed to metalworking fluids to identify any additional cases and *b*) testing of all metalworking fluid reservoirs in the facility for mycobacteria. In addition, the disease occurrence was reported to the Division of Respiratory Disease Studies of the National Institute for Occupational Safety and Health (NIOSH) and the New York State Health Department Occupational Lung Disease registry.

## Discussion

Metalworking fluids are widely used where metal is cut, drilled, milled, or otherwise shaped with cutting tools, to remove heat from both the machine tool and the product being made and to lubricate the parts, remove metal debris, and inhibit metal corrosion. Hypersensitivity pneumonitis is a serious environmental immunologic lung disease in which recurrent exposures to inhaled antigens lead to immunologic sensitization with a predominantly cell-mediated lung response. Subsequent exposures then cause an inflammatory response in the lung that can produce symptoms of dyspnea, cough, and wheeze; fever and elevated blood white count; and transient lung infiltrates and hypoxemia. Persistent disease can cause permanent loss of lung function and even death. Many patients develop disease from exposures associated with work, although exposure to biologic aerosols from home can also cause disease (Apostolakos et al. 2001; Kawai et al. 1984; Wright et al. 1999).

Hypersensitivity pneumonitis was first described in dairy farmers exposed to aerosol from stored, moldy hay containing mixed microorganisms. The list of inhaled substances or mixtures known to cause this condition has grown over the years (Patel et al. 2001); most (but not all) causative agents are biologic materials, including proteins from pigeons and other domestic birds. Blood tests for serum precipitating antibodies to a panel of approximately 10 common causes of hypersensitivity pneumonitis are available from commercial laboratories. However, disease may occur from exposure to substances not included in these panels. In addition, exposure may result in asymptomatic sensitization. Use of precipitating antibodies in diagnosis of hypersensitivity pneumonitis is limited by these factors.

Metalworking fluids may be pure petroleum oils (“straight oils”), emulsions of petroleum in a water base (semisynthetic fluids), or emulsions of synthetic oils in water (synthetic fluids). Because they contain biologically available carbon (in the form of lipids) and water, water-based metalworking fluids routinely sustain microbial growth, but excess growth degrades the fluids and leads to loss of usefulness. Thus, standard use of these metalworking fluids in industry often includes routine testing for bacteria counts (without identification of all organisms) and the use of microbicides with the objective of suppressing, although not necessarily sterilizing, microbial growth.

A variety of respiratory illnesses have been reported to be associated with occupational inhalation of metalworking fluids, including bronchitis, asthma, and lipoid pneumonia (Cullen et al. 1981; Kennedy et al. 1989; Leigh and Hargreave 1999), and their toxicology has recently been reviewed (Gordon 2004). Currently there is no specific Occupational Safety and Health Administration (OSHA) standard for metalworking fluids, although guidance in prevention of health hazards is provided in an NIOSH criteria document [Centers for Disease Control and Prevention (CDC) 1998]. An advisory panel appointed by OSHA recommended a new permissible exposure limit of 0.4 mg/m^3^ thoracic particulate and 0.5 mg/m^3^ total particulate (OSHA 1999), based in large part on the NIOSH criteria document. However, at present, this recommendation has not been the subject of rule making. Hypersensitivity pneumonitis associated with metalworking fluids was first described in 1995 (Bernstein et al. 1995). Since then, numerous outbreaks have been described, associated with inhalation of aerosols of water-containing metalworking fluids (reviewed in Kreiss and Cox-Gaenser 1997). Prevention efforts have focused on reduction of inhalation exposures by workplace modifications that reduce generation of aerosols or improve dilution and ventilation of workplace air, and one follow-up study has documented successful remediation (Bracker et al. 2003).

More recently, outbreaks of this condition have been found in workplaces with metalworking fluids containing nontuberculous mycobacteria (CDC 2002; Kreiss and Cox-Gaenser 1997), most frequently *M. immunogenum*. Detection of these mycobacteria requires special laboratory culture and identification techniques that are not included in routine microbiologic testing of industrial metalworking fluids, such that their identification requires knowledge of their potential for growth and the ability to perform special testing.

During recent years, association of hypersensitivity pneumonitis disease with a different species, *Mycobacterium avium* complex (MAC), from hot tubs, whirlpool baths, and spas has also been identified, sometimes referred to as “hot tub lung” (Capelluti et al. 2003; Grimes et al. 2001; Rickman et al. 2002; Scully et al. 1997). In these hot water bathing tubs, water may be agitated by powerful jets of air or water that produce bubbles and hence aerosols of water droplets. MAC grows well in the high water temperature of the indoor hot tub. The combination of MAC organisms’ growth and jet aerosolization and subsequent inhalation of large amounts of MAC presumably leads to the development of this disease. Hot tub lung appears to be hypersensitivity pneumonitis to MAC aerosol rather than a direct infection of the lung, although this subject is still a matter of debate (Aksamit 2003; Embil et al. 1997). Interestingly, there have been no documented cases of hot tub lung with outdoor hot tubs.

In hot tub lung, pulmonary function tests were mainly restrictive with occasional obstruction (Anonymous 2000; Kahana and Kay 1997; Khoor et al. 2001; Mangione et al. 2001; Mery and Horan 2002; Rihawi et al. 2004). Chest radiography shows diffuse infiltrates, and high-resolution CT of the chest shows ground glass opacities and micronodules (Pham et al. 2003). Sputum culture was positive for MAC in about 70% of the patients; transbronchial biopsy and bronchoalveolar lavage cultures increased the yield further (Anonymous 2000; Kahana and Kay 1997; Khoor et al. 2001; Mangione et al. 2001; Mery and Horan 2002). Hot tub water usually grows MAC. The histopathologic findings reveal discrete nonnecrotizing granulomas with centrilobular and bronchiolocentric distribution. The granulomas described in hot tub lung were more exuberant and well formed than those seen in typical cases of hypersensitivity pneumonitis from other causes.

There is no standard approach to treatment of hot tub lung. Case reports describe significant improvement with removal from exposure to the hot tubs. Oral corticosteroids, antimycobacterial therapy, or both have also been used. The expected course of this disease after the above measures is recovery without relapse. Measures proposed as being helpful in prevention include better ventilation of the hot tub room, frequent cleaning of the hot tub, frequent change of hot tub water, and use of disinfectants such as chloramines, bromine, and ultraviolet light. These measures are similar to those usually proposed for prevention of hypersensitivity due to exposure to myco-bacteria in metalworking fluids.

## Conclusions

Environmental mycobacteria have been associated with a serious lung condition, hypersensitivity pneumonitis, when inhaled as part of liquid droplet aerosols generated from large volumes of liquids serving as a culture medium. These organisms are found commonly in nature and are able to grow in sufficient quantities to cause disease. The case reported here involved an occupational source of such an exposure (aerosolized metalworking fluid in a machining environment), although aerosols containing mycobacteria have been described in other settings as well (aerosolized water from hot tubs). For this reason, specific investigation of sources of aerosols in the work or home environment of patients with this condition should consider the growth of mycobacteria as one of the potential sources of disease. As with other causes of hypersensitivity pneumonitis, removal from exposure and remediation of exposure are the first approaches to treatment.

## Figures and Tables

**Figure:**
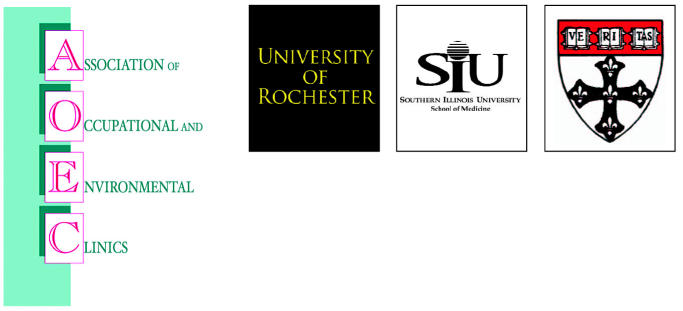
The University of Rochester Southern Illinois University The Harvard School of Public Health

**Table 1:** Patient’s pulmonary function laboratory data.

				O_2_ saturation (%)		
Date	FEV_1_ (%)	FVC (%)	D_L_CO (%)	Rest	Exercise	Notes
June 1985	2.70 (88)	3.0 (70)	—	—	—	Preplacement work exam before onset of symptoms
January 1996	2.77 (94)	3.47 (95)	—	—	—	After onset of symptoms; spirometry before the work week
January 1996	2.98 (101)	3.40 (93)	—	—	—	After shift at end of work week
September 1997	—	—	—	96	96	—
January 1998	—	—	(67)	—	—	—
April 2000	2.52 (89)	3.14 (89)	9.8 (44)	92	89	More symptomatic
June 2000	1.86 (60)	2.55 (65)	—	—	—	—
April 2004	2.42 (89)	3.15 (92)	11.5 (45)	—	—	Symptoms stable

Abbreviations: —, not measured; D_L_CO, diffusing capacity for carbon monoxide (percent predicted); FEV_1_, forced expiratory volume in 1 sec in liters (percent predicted); FVC, forced vital capacity in liters (percent predicted).
